# Plant Feed Additives as Natural Alternatives to the Use of Synthetic Antioxidant Vitamins on Yield, Quality, and Oxidative Status of Poultry Products: A Review of the Literature of the Last 20 Years

**DOI:** 10.3390/antiox10050757

**Published:** 2021-05-11

**Authors:** Rosario Pitino, Massimo De Marchi, Carmen L. Manuelian, Marion Johnson, Marica Simoni, Federico Righi, Eleni Tsiplakou

**Affiliations:** 1 Department of Veterinary Science, University of Parma, Via del Taglio 10, 43126 Parma, PR, Italy; rosario.pitino@unipr.it (R.P.); marica.simoni@unipr.it (M.S.); 2 Department of Agronomy, Food, Natural Resources, Animals and Environment, University of Padova, Viale dell’ Università 16, 35020 Legnaro, PD, Italy; massimo.demarchi@unipd.it; 3 BHU Future Farming Centre, P.O. Box 69113, 7640 Lincoln, New Zealand; marion.johnson@bhu.org.nz; 4 Laboratory of Nutritional Physiology and Feeding, Department of Animal Science, School of Animal Biosciences, Agricultural University of Athens, Iera Odos 75, GR-11855 Athens, Greece; eltsiplakou@aua.gr

**Keywords:** organic, plant extracts, antioxidant vitamins, tocopheryl, tocopherols, poultry

## Abstract

Scientific evidence demonstrates that plant feed additives (PFA) can be a viable alternative to synthetic antioxidant vitamins in poultry nutrition. PFA are represented by plants, essential oils, plant extracts, and by-products from herbal or crop processing. The use of PFA in the feed industry has increased in recent years as their biologically active compounds (polyphenols) have demonstrated antimicrobial and antioxidant effects in food-producing animals. However, few trials have directly compared the effects of PFA with synthetic vitamins. After a systematic literature review of studies comparing the effects of PFA and synthetic vitamins on poultry products in the last 20 years (2000–2020), a total of 44 peer-reviewed articles were included in the present work. A positive effect of PFA on poultry products’ oxidative stability during storage, organoleptic characteristics, and fatty acids profile has been observed without a specific impact on their performances. The effects of PFA are variable but often similar to those of vitamin E, suggesting the opportunity for a partial substitution of the latter in poultry diets.

## 1. Introduction

The growing consumer desire for safe and natural foods of animal origin and the European ban of in-feed antibiotics in animal nutrition have increased the interest in the use of plant feed additives (PFA) as a dietary ingredient in animal nutrition [1,2,3]. PFA are represented by a wide group of plant tissues, plant extracts, essential oils, and plant by-products containing biologically active compounds with potential positive effects on animal productivity [4,5]. Many PFA have been demonstrated to express significant antioxidant activity, potentially improving animal production and the oxidative status of both the organism [6] and product. The antioxidant components of PFA belong to different chemical classes of plant secondary metabolites which may be included in animal diets as extracts (plant extracts), essential oils, or resins and are characterized by a significant content of bioactive molecules [7,8].

From PFA, it is possible to insulate polyphenols—flavonoids being the main group that comprises several molecules of interest for animal feeding—phenolic acids, and stillbenoids [9]. Polyphenols are bioactive compounds capable of interacting with feed matrices, reducing or enhancing the accessibility and availability of nutrients. The metabolized product generated after their absorption is the main bioactive component that enters the systemic circulation and reaches the targeted organs [10]. Moreover, from each PFA, it is also possible to extract individual active components, thus reducing the dose of supplement to be administered, and avoid some issues related to odor and taste [11]. Besides the potential detrimental organoleptic characteristics, several factors may negatively influence the effectiveness of feeding essential oils or plant extracts to animals. Some are related to the plant harvesting phase, while others are linked to the interaction with the feed matrix, intestinal bioavailability, and the mode of action [3]. Moreover, a variety of factors influencing the antioxidant activity of some PFA rich in polyphenols need to be considered. For example, it has been emphasized that the absorption of flavonoid compounds at the intestinal level is poor, which reduces their concentration in the target tissue where the antioxidant effect should be exerted [9]. Despite these controversial aspects, PFA have been widely tested in poultry in an effort to maintain a high level of growth performance and other feed efficiency parameters, as well as increasing the quality of poultry products, ameliorating negative carcass traits, and delaying lipid oxidation processes in breast and thigh meat [1].

A number of studies have been conducted to evaluate the potential of PFA as vitamin sources, hence the requirement for a summary of the published scientific evidence on the impact of the use of PFA on poultry productivity and poultry food product quality. The influences of PFA on poultry’s feed intake, feed efficiency, growth traits, and nutrients digestibility; on metabolic and hematological parameters and antioxidant status; on macro- and microscopic anatomical features; and on the intestinal microbiome profile have already been presented by Righi et al. [6]. Therefore, the present review focuses more detail on the antioxidant capacity of plant extracts (PE), essential oils (EO), and by-products of plant origin (BP) as potential alternatives to synthetic vitamins in poultry-derived food products’ yield, quality, and oxidative status.

## 2. Materials and Methods

A systematic review of peer-reviewed studies published in Pubmed (www.ncbi.nlm.nih.gov; last accessed on 10 January 2021), ISI Web of Science (www.webofknowledge.com; last accessed on 10 January 2021), and Sciencedirect (www.sciencedirect.com; last accessed on 10 January 2021) databases was performed covering the 20 years between January 2000 and December 2020. The keywords used for the search were: plant extract, plant by-product, essential oil, natural vitamins, synthetic vitamins, poultry, vitamin E, vitamin C, tocopherols, tocopheryl, antioxidants, and organic farming. Documents were selected firstly based on the title and then on the abstract. If the objective of the work and the tested parameters were in agreement with the selection criteria and consistent with the aim of the present review, the articles were downloaded and summarized in tables that were then described and discussed in the text. The selection criteria were as follows: (i) articles comparing the effect of PE or EO with a specific dose of synthetic vitamins or synthetic antioxidants in poultry; (ii) articles comparing the effect of BP from different agro-industries with a specific dose of synthetic vitamins or synthetic antioxidants in poultry; (iii) articles published in peer-reviewed journals; and (iv) exclusion of studies dealing with propolis, algae, and additives of animal origin. At the end of the selection, a total of 44 papers were retained for the review, of which 35 dealt with meat products and 9 focused on eggs. Of the 44 studies, 17 were conducted in Greece, and 19 papers compared rosemary extract/oil and/or oregano extracts/oil with vitamin E (VitE). The present review is divided into two sections (i.e., meat and eggs production) and according to the group of parameters evaluated: (i) slaughter traits; (ii) meat quality characteristics; (iii) lipid oxidation of poultry meat; (iv) microbial contamination; (i) egg production yield and traits; and (ii) lipid oxidative stability of eggs. 

## 3. Potential Plant Extracts and Plant By-Products as Alternative Sources of Vitamins for Animal Feeding: Impact on Productivity

### 3.1. Meat Production 

The effect of PFA on meat is relevant for both food safety and economic reasons. Several studies have tested the use of PFA in poultry diets to evaluate their impact on slaughter traits and meat quality parameters, including lipid oxidation indexes, and compared them with synthetic antioxidants such as VitE (Table S1).

#### 3.1.1. Slaughter Traits (Carcass Weight and Quality, and Internal Organ Weights)

The PFA effects on slaughter traits have been investigated in some articles, as a consequence of in vivo growth performances. It should be noted that the possible effects on these traits could be attributable to the modulation that PFA exert on the balance of intestinal microbiota [12].

The inclusion of hesperidin in the diet of broilers (HE, 0.15 or 0.3%) did not affect intramuscular fat (%), cooking loss (%), shear values, cold carcass weight (CCW), or the weights of the liver, heart, gizzard, and abdominal fat (% of CCW), when compared with control or with VitE (200 mg/kg)-fed groups [13]. Carcass quality parameters were not modified in broilers supplemented with different herbal additives (rosemary leaves (RL), rosehip, chokeberry pomace, and nettle at 2.5% each of the diet), rosemary powder ((RPO) 0.5, 1.0, 1.5% of the diet, or 1% RPO plus VitE at 100 mg/kg) [14], and a commercial polyphenol product (at 0.2% of the diet) [15]. The same results were observed in broilers and ducks supplemented with different natural antioxidants [16] compared with VitE-fed animals (100 or 200 mg/kg of the diet). Moreover, carcass yield (CY) did not differ in a feeding trial where oregano oil (OO) [17], rosemary oil (RO) [17,18], and RL [18] were compared with VitE. Similarly, no significant effects were observed in a 20-day trial in broilers fed with a basal diet including VitE (150 mg/kg), or either thyme extract (TE), sage extract (SE), coneflower extracts (CE; at 0.056% of the diet), marigold xanthophylls (MX, 0.02% of the diet), a mix of synthetic antioxidants (at 0.0486% of the diet of butylated hydroxytoluene, ethoxyquin, butylated hydroxyanisol), or β-apo-8-carotenoic acid ethylester (0.0040% of the diet) [19]. 

Dietary supplementation with a polyphenol product (PP, 0.01% of the diet) alone or in combination with VitE (PPE, 0.01% of PP plus 100 mg of VitE/kg) in heat-stressed (HS) broilers (Proviox, Provimi, France) improved their breast muscle yield (by 7.4%) compared with those fed a basal diet without VitE or with VitE (100 mg/kg). However, the color parameter referring to yellowness (b*) was lower in both PPE (by 11.8%) and PP (by 13.2%) groups than in broilers reared in a thermoneutral (TN) environment. Natural drip loss and water holding capacity (WHC) were lower in the PPE (by 39.9 and 29.7%, respectively) and the PP (by 37.3 and 24.9%, respectively) than in the HS group. On the other hand, crude ash content was higher in the PP than in the TN (by 9.8%) and the HS group (by 6.0%) [20]. In another similar study, broilers supplemented with the same PP (at 0.02% of the diet) tended to have a higher carcass dressing-out percentage (by 3.4%) compared with the VitE (100 mg/kg)-fed group [15]. The weights of carcasses and organs were not affected by feeding experimental diets; only the gizzard weight was lower (by 15.6%) in the PP than the VitE-fed broilers [12]. Additionally, PP administration alone (at 0.02% of the diet) or in combination with VitE (PPE) in broilers fed with a contaminated grain diet decreased their carcass dressing-out percentage (by 3.8 and 2.6%, respectively), in comparison with those fed an uncontaminated diet (NCD). Broilers in the PP group had heavier livers (by 27.0%) compared with the NCD group, whereas both the PPE and PP groups had longer small intestines (by 22.3 and 24.2%, respectively) and lower small intestine weights (by 20.7 and 25.5%, respectively) when compared with the NCD group. The PPE-fed animals, when compared with those administered with VitE only (100 mg/kg), had lighter small intestine weights (by 9.8%), but shorter ceca (by 13.3%), than those receiving the contaminated diet [15]. The inclusion of thyme oil (TO) (0.01% of the diet) in broiler diets in comparison with a VitE-deficient dietary treatment reduced the hot carcass weight (by 4.1%) [21]. A significant increase (by 3.9%) in hot carcass weight (HCW) was found when the broiler diet was supplemented with TO (at 0.02%) compared with VitE (100 mg/kg), whereas a decrease in HCW (by 4.7%) was observed at lower levels of TO (0.01% of the diet) or higher levels of VitE (200 mg/kg) inclusion [21]. Liver weights of TO-fed broilers were lower (on average, by 12.0%) than those receiving VitE (200 mg/kg) [19]. 

A greater pancreas and liver weight in the RL group than in the control (by 21.5 and 18.6%, respectively) and VitE groups (by 27.6 and 18.2%, respectively) has been reported [22]. In grape pomace (GP, 1.5%)-fed broilers, a greater (by 18.4%) relative weight (RW) of the abdominal fat compared with those consuming a deficient VitE diet was indicated [23]. The same birds showed higher spleen relative weights (RW; by 20%) and a greater relative length (RLE) of the duodenum (by 7.4%) and ceca (by 10.4%), compared with those consuming a VitE (200 mg/kg)-enriched diet [23]. A reduction in heart and liver RW (by an average of 31.3 and 3.5%, respectively) in broilers supplemented with a low amount of grape polyphenols in combination with VitE (25 mg/kg of GPP + 75 mg/kg of VitE) compared with broilers supplemented with VitE (100 mg/kg) only was observed [24]. In a more recent study, grape pomace supplementation (from 5% to 10%) did not affect broilers’ relative organ and carcass weights, nor did supplementation with VitE (200 mg/kg) [25]. From the above studies, it appears that dietary PFA, in comparison to both a basal or a VitE-supplemented diet, can exert variable effects on carcass yield and quality and the weight of internal organs depending on the nature of the product tested, the dose employed, and the presence of stressors possibly affecting animal performance.

#### 3.1.2. Meat Quality Characteristics (pH at 24 h Postmortem, Color, Shear Force Value, Cooking Loss, Intramuscular Fat, Sensory Qualities, Fatty Acid Composition)

Meat quality characteristics are of great importance from a consumer perspective. In many papers, they have been evaluated alongside other variables, such as slaughter traits and the intrinsic characteristics of meat. 

The addition of different PFA at differing dietary inclusion levels did not affect meat color in poultry [12,15,23]. Moreover, PFA did not affect meat pH at 24 h post-mortem (pH24) in broilers and ducks [16]. The administration of sweet chestnut wood extract (SCW) to a basal diet did not modify pH, drip loss, and electrical conductivity of broiler meat [26]. Dietary supplementation with hesperidin (HE; at 0.3% of the diet) increased the pH24 compared with the basal diet, without differences from the VitE (200 mg/kg of the diet)-fed animals [13]. The dry matter, ether extract, and saturated fatty acid (SFA) content in HS broiler breast meat was not affected by the dietary inclusion of PP alone or in combination with VitE (PPE) compared with unsupplemented or VitE-supplemented animals or with those without heat stress [20]. 

Yellowness (b*) in the pectoralis major (PM) and ileotibialis (IL) muscles was improved in broilers consuming oregano plant (OP) compared with basal (by 41.4 and 56.7%, respectively) and VitE (by 51.9 and 74.1%, respectively)-enriched diets for 42 days [27]. Similarly, skin b* increased in nettle-supplemented diets compared with basal (by 90.7%) and VitE (by 77.6%) diets [22]. Lightness (L*) was also improved by 4.5% when broilers were fed with HE (0.15% of the diet) compared to VitE diets [13]. Broilers supplemented with RO (at 0.01, 0.015, 0.02% and VitE at 200 mg/kg of the diet) showed a greater pH24 in breast (by 1.2% on average) and thigh (on average, 0.68%) meat compared with RL-fed ones [18]. Muscle from broilers reared under HS had a greater ash content in the polyphenol- and VitE (200 mg/kg)-supplemented groups than in the negative (no HS and no supplementation) and VitE (100 mg/kg) control groups [20]. Moreover, PP-fed animals had a greater ash content than the positive (by 5.7%) and negative (by 9.8%) control ones. The PP- and low VitE (100 mg/kg)-supplemented animals had a greater polyunsaturated fatty acids (PUFA) content compared with the positive control group (only heat stress). In addition, lower natural drip loss was found in the PP- and VitE (200 mg/kg)-fed animals compared with the basal and low-VitE (100 mg/kg) diets [20].

In another similar trial, dietary supplementation with a combination of VitE (100 mg/kg) and PP (at 0.01 and 0.02%) increased (by 5.1 and 3.8%, respectively) broiler breast muscle pH at 15 h after slaughter in comparison with the basal diet, but not when compared with a diet containing VitE and low-quality oil [28]. On the other hand, the pH decreased (by 2.7%) 24 h after slaughter in poultry consuming the PP diet (at 0.02%) in comparison with the VitE (200 mg/kg) one [26]. Supplementation of PP had positive effects on color parameters. Birds in the PPE and PP groups had an improved L* value compared with the negative (by 3.5 and 5.0%, respectively) and positive control (by 3.7 and 5.2%, respectively) groups. Similarly, the L* value was higher in the PP group than in VitE groups (by 4.8%, on average). However, the other color parameters were not affected. With respect to the fatty acid composition of breast muscle (BM), dietary supplementation with PP alone or in combination with VitE did not exert a positive effect on PUFA, hypocholesterolemic fatty acids (DFAs), neutral and hypocholesterolemic/hypercholesterolemic fatty acids ratio, or on the atherogenicity index, unlike VitE [28]. When broilers were fed experimental diets containing ochratoxin A and with the same doses of antioxidants given in the previous trial, PP and VitE were not effective in reducing the detrimental effect of the contaminated diet. The percentages of SFA and oxo-fatty acids (OFA) were, in fact, significantly higher, while those of PUFA, n-3 FA, and DFA were lower in the ochratoxin A-fed animals compared with those consuming the uncontaminated diet [15].

The dietary inclusion of OP (3% of the diet), during a 42-day trial, resulted in a lower L* value (by 4%) in broilers’ PM than the inclusion of vitamin C (VitC; 1000 mg/kg of the diet) and VitE (200 mg/kg of the diet) [27]. The addition of HE (0.15% of the diet), compared with VitE, reduced the redness a* (by 15.9%) and b* (by 8.6%) [13]. Therefore, in these two previous studies, color was not improved by feeding PFA. The incorporation of PFA alone or in combination with VitE (0.02% of the diet, PP, or 0.01% + 100 mg/kg of VitE, PPE) in HS broiler diets induced a lower WHC compared with the positive HS control treatment [20]. Despite the greater PUFA content observed in the supplemented groups, lower concentrations of eicosadienoic acid (C20:2), arachidonic acid (C20:4), eicosapentanoic acid (C20:5), and docosapentanoic acid (C22:5) and greater proportions of n-6 fatty acids were observed compared with unsupplemented HS broilers [20]. Conversely, OO inclusion in two different diets (CSB, crude soybean oil diet; ASO, acidulated soybean oil soapstock) did not affect the proportions of SFA, MUFA, PUFA, and the PUFA/SFA ratio in broilers’ BM compared with VitE (10 or 100 mg/kg) administration. However, the proportion of linolenic acid (C18:3) was higher in OO compared with the VitE-fed animals (38.4 and 44.4%, respectively) [29]. 

Supplementing the diet of broilers with oregano aqueous extract (OAE, 0.02% of the diet) did not produce any significant effects on the physicochemical, proximate, and fatty acid (including CLA isomers and PUFA n-3 content) composition of the BM, in comparison with those consuming the control (low VitE content) and the VitE diet (150 mg/kg) [30]. When SE was added to a broiler diet, an increase in the proportions of stearic acid (C18:0) (by 20.1%), arachidonic acid (20:4) (by 44.7%), DHA (C22:6 n-3) (by 59.6%), and n-3 PUFA was recorded in comparison with those fed the basal diet, but not with the VitE-fed ones [19]. In the same trial, the breast meat of the SE group had a lower content of oleic acid (C18:1) (by 12.8%) compared with the control group, and a lower alpha-linolenic acid (C18:3) (by 24.6%), PUFA (by 24.5%), and n-6/n-3 ratio (by 23.4%) compared with animals receiving synthetic antioxidants [18]. 

In general, when compared to either a basal diet or one supplemented with VitE, it appears that PFA were able to increase the pH and ash content of poultry meat and lower drip loss. Moreover, depending on the study, they sometimes improved yellowness (b*) and lightness (L*) and increased the PUFA content.

#### 3.1.3. Lipid Oxidation of Poultry Meat

Despite poultry meat having many nutritional characteristics appreciated by the consumer, the interest in the relatively high content of PUFA in the muscle has led to research on dietary manipulation which may increase the unsaturated fat content of muscle membranes [31]. This change, however, increases the susceptibility of poultry meat to lipid oxidation [32]. Several papers have tested PFA in comparison with different doses of VitE to assess their effectiveness in delaying lipid oxidation in poultry meat samples stored under different conditions. In order to demonstrate the antioxidant activity in meat products, thiobarbituric acid (TBA) analysis is used to measure malondialdehyde (MDA), which is a product of meat oxidation that increases during storage [33].

A significant reduction in MDA values in refrigerated breast (up to 47%) and thigh meat (up to 30.2%) samples was observed in broilers receiving GP (at 1.5, 3, and 6%) compared with those consuming a basal diet [23,34]. The same effect was found in breast (by 22.4%) and thigh (by 62.3%) muscle (raw and warm tissue) when broilers were fed with a commercial blend of essential oils (Apacox^®^, APA-CT, Italy: APA; 0.01 and 0.05% of the diet) [35]. Moreover, addition of HE (0.15 or 0.3% of the diet) in broilers’ diets reduced (on average, by 41.15%) the MDA content in raw pectoralis muscle after 9 days of storage [13]. Dietary supplementation with RL (0.57 to 1.15% of the diet) and RO (0.010 to 0.020% of the diet) for 42 days decreased the MDA content (on average, by 27.3 and 25.5%, respectively) in broiler meat when measured over 5 days post-mortem compared with VitE (50 and 200 mg/kg) supplementation [18]. A combination of essential oils (0.015% of both RO and OO diets) compared with VitE reduced the MDA value (up to 53.7%) and delayed lipid oxidation of breast and thigh samples at the end of the 15 days of storage [17]. The dietary inclusion of OO alone (0.5% of the diet) or in combination with VitE (170 mg/kg) or VitC and VitE simultaneously (190 and 170 mg/kg, respectively) did not affect the MDA values, although the antioxidant capacity of the raw and cooked meat was improved [36]. The administration of RPO (1% of the diet) and VitE (200 mg/kg) reduced MDA values in broiler breast meat after 14 days of preservation compared with the inclusion of different dose rates and additive combinations (RPO, 0, 0.5, or 1.0% of the diet; and/or VitE, 0, 100, or 200 mg/kg) [14]. 

A combination of essential oils (0.015% of both RO and OO of the diet) was as effective as VitE in preserving the sensory qualities of breast meat after 15 days of storage [17]. Moreover, the addition of OO (0.010% of the diet) to a broiler diet was as efficient as VitE (200 mg/kg) in reducing MDA values in samples of breast meat during a 9-month storage period at −20 °C [37]. Similar findings were observed in stored samples of broilers’ IL and liver when their diet was supplemented with OP (3% of the diet) or vitamins (VitC, 1000 mg/kg; and VitE, 200 mg/kg) during a 42-day period [27]. Grape polyphenols (GPP) added at different levels in broiler diets (0.0025 to 0.0075% of the diet), compared with VitE (100 mg/kg), increased free radical scavenging activity in breast and leg muscles (from 4.8 to 14%). Total phenolic content increased (by 20.4 and 17.6%, respectively) and thiobarbituric acid reactive substances (TBARS) decreased (by 18.2 and 16.7%, respectively) in breast and in leg muscle [24,38]. The inclusion of tomato pomace (TP; 30%) compared with VitE in broiler diets decreased the TBARS value of the meat (by 23.0%) after 4 days of storage [39]. 

In turkeys, both dietary supplementation with OO (at 0.2% of the diet) and VitE (200 mg/kg) delayed lipid oxidation in breast, thigh, liver, and heart tissues to the same extent [40]. Moreover, a synergistic effect was observed when OO (0.1% of the diet) and VitE (100 mg/kg) were added simultaneously. By contrast, the addition of sweet chestnut wood extract (SWE, 3% of the diet) to a PUFA-rich diet did not significantly affect the stability of the meat compared with different levels of VitE [26]. 

Some studies indicated that synthetic antioxidants can be more effective in improving the oxidative stability of meat in comparison with PFA. More specifically, GP, in relation to VitE (200 mg/kg), induced higher MDA values (between 32 and 67%), depending on its dietary dose, muscle type, and storage time [23,34]. VitE, compared with a mix of essential oils (Apacox^®^; APA), not only reduced the MDA levels in breast and thigh muscles (raw and heated tissue) but also increased their α-tocopherol concentration [35]. Similarly, dietary VitE supplementation (200 mg/kg) displayed greater (by an average of 329%) antioxidant activity than HE regardless of the supplementation rate in raw pectoralis muscle after 9 days of storage [13]. Although the dietary inclusion of either OO (at 0.1 and 0.2% of the diet) or VitE (200 mg/kg) delayed lipid oxidation in broiler and turkey tissue samples, VitE was more effective in reducing MDA values in the broilers, but not in the turkeys [41,42]. The combination of OO (0.01% of the diet) and VitE (100 mg/kg) gave the best antioxidant synergistic effects, both in frozen and refrigerated turkey meat [42,43]. Supplementation with OP compared with the administration of vitamins (VitC and VitE) was less effective in reducing MDA values in the IL and PM muscles (by 88.3 and 71.8%, respectively) in HS broilers [27]. Although the highest inclusion level of VitE (300 mg/kg) in a turkey diet gave the sharpest decline in the MDA values of their breast meat after 12 days of storage, supplementation with dehydrated rosemary extract (RE at 1% of the diet), RL (1% of the diet), or olive leaves (OLE; 0.5% of the diet) led to better results than or similar results to those from turkeys consuming lower doses of RE (0.5% of the diet) [44] or VitE (150 mg/kg) [45,46]. In turkeys, VitE was also more effective in decreasing MDA levels during 9 days of storage than RL alone (0.5 and 1% of the diet) or in combination with VitE (0.5 or 1% of the diet + 300 mg/kg of VitE) [47]. VitE (200 mg/kg) supplementation led to lower MDA values in the breast and thigh muscles of broilers compared with OP (at 0.5 and 1% of the diet). However, OP seemed more effective than OR (administered at the same doses) in reducing MDA values [48]. Dietary supplementation with TO (at 0.01 or 0.02% of the diet) affected lipid oxidation of leg and breast muscles of broilers [21]. In leg muscle, after 7 days of storage, TO consumption reduced TBARS values (average by 97.0%) compared with the control group, whereas TO consumption (0.02% of the diet) decreased TBARS compared with low- (by 20%) and high (by 33.3%)-VitE administration. Similar results were observed in breast muscle, in which TO administration lowered the TBARS values (by 96.8%) compared with the basal diet. Additionally, TO was more effective (on average, by 27.5%) in delaying lipid oxidation but only in comparison with the lower (100 mg/kg) inclusion level of VitE [21], whereas VitE (200 mg/kg) and TO (0.02%) were equivalent in delaying lipid oxidation in breast muscle [21]. 

Conversely, greater TBARS values in the meat were found when broiler diets were supplemented with natural tocopherols (NT), rosemary extract (RE), green tea extract (GTE), grape seed extract (GSE), or tomato extract (TOE) (0.01 and 0.02% of the diet) in comparison with synthetic antioxidants (SYNT; 300 mg/kg, including butylated hydroxytoluene, ethoxyquin, butylated hydroxyanisole, and vegetable oil) alone or in combination with VitE (200 mg/kg; SYNT + VitE). No synergistic effects on TBARS values were observed. However, the higher rather than lower inclusion levels of NT, GSE, and TOE led to a noticeable reduction in the TBARS value [49]. Although VitE (200 mg/kg) was more effective in reducing TBARS values and increasing the resistance against oxidation of the abdominal fat in broiler meat samples after 9 days of storage, RL administration was able to reduce (by 57.2%) and increase (by 32.6%) the above parameters, respectively, in comparison with the basal diet [22]. Moreover, VitE (150 mg/kg) was more effective than marigold xantophyll (MX; 0.002% of the diet), CE, TE, and SE (0.056% of the diet each) in reducing TBARS values (by 25.2%) and delaying lipid oxidation in broiler meat samples [19]. Tea catechins from green tea leaves (GTL, Camellia sinensis; 0.3% of the diet) significantly delayed lipid oxidation to the same extent as VitE (200 mg/kg) in frozen chicken thigh meat for up to 9 months of storage [50]. In another study by the same authors, it was found that catechins (at 0.3% of the diet) were superior to VitE (200 mg/kg) in improving the oxidative stability of chicken thigh meat [51]. Conversely, dietary GTL compared with VitE (200 mg/kg of the diet) increased TBARS values (by 213%) in thigh meat [16]. The dietary addition of tomato skin and orange peel (0.2% each), for 56 days in broiler diets, was as efficient as VitE (200 mg/kg) in reducing TBARS levels in leg meat. In ducks, orange peel and RL decreased TBARS values (by 71.0 and 33.0%, respectively) in breast meat, but not as much as VitE (by 82%), compared with the basal diet; however, in legs, orange peel and RL supplementations were at least as effective as VitE in reducing (by 66.0, 47.0, and 46%, respectively) TBARS values [16]. Therefore, dietary supplementation with GP, HE, APA, OO, OP, and RO can reduce MDA levels in poultry samples under refrigeration, and sometimes as effectively as VitE. In some cases, a synergistic effect between PFA and synthetic vitamins (VitE and VitC) was reported, allowing a reduction in the levels of synthetic vitamins added to the diets of poultry. 

Dietary supplementation with PP alone (0.02%) or in combination with VitE (0.01% of PP + 100 mg/kg of VitE) in comparison to receiving only an ochratoxin-contaminated and low-antioxidant diet decreased TBARS values (by 55.3% and 42.6%, respectively) in broiler breast muscles [15]. However, this difference in TBARS values was not observed when the comparison was made with VitE supplementation [12]. In another trial, consumption of the same product (at 0.02% of the diet) did not affect TBARS values in the BM of broilers fed with low-quality oil [28]. On the contrary, the TBARS levels were decreased (by 82.7%) when the animals were fed PP and VitE simultaneously.

In summary, many in vivo studies demonstrate that PFA dietary inclusion can delay lipid oxidation in poultry meat under different storage intervals. These effects were often lower than those exerted by VitE or at least equivalent when PFA were fed at the highest doses. Moreover, a synergistic effect between PFA and synthetic vitamins has been shown, introducing the possibility of reducing the inclusion level of synthetic vitamins in poultry diets when needed, as in the case of organic livestock systems.

#### 3.1.4. Microbial Contamination of Poultry Meat

The addition of PFA has also been demonstrated to affect the microbial contamination of meat. For instance, dietary supplementation with RL and OL showed an inhibitory effect on microbial growth in turkey meat compared with the basal and VitE (150 mg/kg or 300 mg/kg) treatments [45]. The administration of VitE (at 50 mg/kg and 200 mg/kg) was more effective than RL (by 43.2%) and RO (by 36.3%) in controlling *Escherichia coli* proliferation on poultry meat [18]. In turkeys’ meat, RE (0.5 and 1% of the diet) compared with VitE (300 mg/kg) and basal diet consumption reduced the total viable bacteria count and showed a greater inhibitory effect on the growth of the other bacterial species after 12 days of storage [44]. Similar results were also observed when adding OL in the turkeys’ diet [46].

### 3.2. Egg Production 

#### 3.2.1. Egg Production Yield and Traits

The literature examining the effects of PFA on egg production is limited and, at times, contradictory. Only a small number of trials have compared the use of PFA with a specific dose of synthetic vitamins (Table S2).

Dietary supplementation with RP (at 0.5 and 1% of the diet), OP (at 0.5%), saffron plant (at 2%), GTE (0.5% of the diet), green tea powder (GTP, 1.5% of the diet), and marigold (0.5 extract; 1.5% powder) and roselle calyx (crude extract, 1 and 2% of the diet; and powder, 2 and 4% of the diet) compared with VitE (200 mg/kg) did not affect the characteristics of eggs (e.g., egg weight, egg shape index, yolk diameter, yolk height, yolk color, albumen height, Haugh units, shell thickness, egg production, specific gravity, and pH of yolk and albumen) or TBARS values when eggs were either refrigerated or at room temperature [52,53,54,55,56]. Moreover, the cholesterol and triglyceride content in eggs from laying hens supplemented with marigold and green tea did not differ from VitE-fed animals [54]. In Japanese quail, the dietary inclusion of anise seed (AS; 1, 2% of the diet) compared with VitE (600 mg/kg) affected neither egg production nor egg quality features [57]. Only changes in the egg yolk color were observed. In fact, egg yolk was less light in the AS group (on average, by 4.1%), while a trend for a higher redness (a*) was observed in the VitE (average of 79%) group in comparison with AS-fed animals [50]. However, the dietary incorporation of marigold powder (MPO) compared with VitE significantly decreased egg mass (by 11.1%), while GTP and GTE reduced yolk weight (by 4.6%) in laying hens [54]. Moreover, the yolk index was not improved with MP, GTP, and GTE compared with VitE [54]. On the other hand, feeding MPO (at 1.5% of the diet) led to an increase in yolk cholesterol content compared with the other dietary treatments and particularly compared with VitE (by 7.1%) [50]. From the available data, it appears that only a small number of changes, mainly to egg mass and yolk weight, are induced by the use of PFA in egg production in comparison with VitE supplementation. However, the limited literature on the topic does not allow the drawing of specific conclusions. 

#### 3.2.2. Lipid Oxidative Stability of Eggs

The oxidative stability of eggs is of great importance for consumers as a higher stability implies a longer shelf life. Trials have explored the potential of PFA, in comparison with synthetic antioxidants, to extend shelf life by delaying lipid oxidation. However, the literature comparing the effectiveness of PFA compared to a positive control group including Vitamin E is still very limited.

In laying hens, dietary supplementation with RE (0.05 and 0.1% of the diet) compared with VitE (200 mg/kg) for 25 days did not affect the lipid oxidative stability of n-3-fatty acid (FA)-enriched eggs [58]. The lowest lipid oxidation rate (MDA) in eggs was observed in VitE (200 mg/kg)-fed laying hens in comparison with those consuming RP (at 0.5 and 1% of the diet) [52]. An opposite result was reported in another study, in which the greatest lipid oxidation in yolk was observed in VitE (200 mg/kg)-fed laying hens [53]. RP supplementation showed a dose-dependent antioxidant activity [52], while SP showed better oxidative stability than RP and OP administrations. On the other hand, dietary supplementation with olive leaves (OL, at 1% of the diet) compared with VitE (200 mg/kg) did not affect fatty acid composition, MDA, and lipid hydroperoxide levels in fresh eggs [59,60]. Moreover, dietary inclusion of OL and VitE did not protect fresh eggs from lipid oxidation compared with a basal diet, while dietary supplementation with VitE compared with OLE lowered TBARS values [60]. From the above studies, it appears that the tested PFA are not always able to reduce the susceptibility of eggs to oxidative processes, and, when successful, they presented a lower potential than VitE, even if their impact could be related to the dose tested and to the diet/eggs characteristics (i.e., unsaturated fatty acids content). 

## 4. Final and General Remarks

In the present review, the available studies regarding the use of PFA and the consequent impact on poultry products’ yield and quality were summarized. The main focus was on the antioxidant capacity of PFA as potential alternatives to synthetic vitamins and, in particular, on their effect on the characteristics of poultry products. As presented in Righi et al. [6], the inclusion of PFA as antioxidants in poultry diets can be effective in mitigating oxidative stress in vivo and in improving productivity and efficiency. Consistently with these conclusions [6], some post-mortem studies confirmed the capacity of grape pomace to reduce lipid peroxidation of poultry meat during refrigerated storage, with no effects on organ weight. A similar effect was exerted by oregano essential oil and oregano oil, the antioxidant efficiency of which was similar to or sometimes higher than VitE. The same PFA prevented a drop in VitE in tissues during preservation. Rosemary oil and rosemary plant alone or in combination with olive leaves or oregano essential oil reduced MDA and inhibited microbial growth during storage, at the same time exerting a sparing effect on VitE. However, the PFA effects were, in general, less pronounced than the VitE ones in poultry products. Hesperidin was also effective in reducing lipid oxidation in comparison with VitE, even when used in a lower amount. Tea catechins reduced TBARS values in poultry meat, but with a lower efficiency than VitE, while rosemary leaves and orange peel were more efficient at reducing TBARS levels than VitE. Coneflower, thyme, sage, and marigold extracts, along with sweet chestnut wood extract, increased the long-chain polyunsaturated fatty acid content of breast muscle. In addition, sweet chestnut wood extract improved meat color more than VitE, while organoleptic meat traits were significantly improved by the dietary addition of oregano aqueous extract. Thyme oil decreased carcass and organ weights while reducing TBARS values more effectively than VitE in a dose-dependent manner. 

Moreover, rosemary plant was more effective in reducing lipid peroxidation of eggs than VitE. The same effect was obtained by feeding olive leaves to the animals. Olive leaves generally increased long-chain polyunsaturated fatty acids in eggs, while a lowering of cholesterol and triglyceride levels in yolk was demonstrated by the addition of green tea and marigold powder/extract to diets. 

## 5. Conclusions

Consistently with in vivo study findings, the inclusion of PFA in poultry diets exerts positive effects on product quality, improving oxidative stability and preservation as well as all the related traits (odor, color, fatty acids, etc.), without a specific impact on product yields. Compared with the synthetic antioxidant VitE, the effects of PFA are variable but often similar, suggesting their possible use, at least in partial substitution of synthetic VitE, in poultry diets.

## Data Availability

The data presented in this study are available free of charge for any user on request from the corresponding authors.

## Supplementary Materials

The following are available online at https://www.mdpi.com/article/10.3390/antiox10050757/s1, Table S1 Effects of plant feed additives on slaughter and meat traits in poultry, Table S2 Effects of different plant feed additives on quantitative and qualitative traits of egg production.
