# Supplementary material for: Plant Feed Additives as Natural Alternatives to the Use of Synthetic Antioxidant Vitamins on Yield, Quality, and Oxidative Status of Poultry Products: A Review of the Literature of the Last 20 Years
Source: Antioxidants (Basel). 2021 May 11;10(5):757. doi: 10.3390/antiox10050757 (PMC8151085; doi:10.3390/antiox10050757)
Supplement: Supplementary file 1 [file antioxidants-10-00757-s001.zip › antioxidants-1175101-supplementary.pdf]

Table S1. Effects of plant feed additives on slaughter and meat traits in poultry

PFA	Dose extract (% of the diet)	Vitamin compared	Type of parameter	Trait evaluated	Comparison to negative control: effect (plant product dose)	Comparison to positive control: effect (dose)	Period of study (d, days, wk, week)	Reference
**Grape pomace**	1.5, 3, 6	E, 200 mg/kg	Lipid oxidation	MDA (1 d)	↓13.64% (1.5%) ↓31.82% (3%) ↓4.91% (6%)	↑26.7% (1.5%)	21 d	Brenes et al., (2008)
				MDA (4 d)	↓28.13% (3%)	↑25% (1.5%)		
				MDA (7d)	↓8.33% (1.5%) ↓27.8% (3%) ↓36.1% (6%)	NS		
			Organ weights	Relative weight (RW) of abdominal fat	↑18.42% (1.5%)	NS		
				RW of liver, pancreas	NS	NS		
				RW of spleen	NS	↑20% (1.5%)		
				RLE duodenum	NS	↑7.41% (1.5%)		
				RLE of jejunum, ileum	NS	NS		
				RLE of ceca	NS	↑10.39% (1.5%)		
**Grape pomace**	0.5, 1.5, 3	E, 200 mg/kg	Lipid oxidation during refrigerated storage	MDA breast (1 d) breast	NS	↑44.4% (1.5%)	21 d	Goñi et al., (2007)

				MDA breast (4 d) breast	↓30% (1.5) ↓33.3% (3%)	↑20.83% (0.5%)		
				MDA breast (7 d) breast	↓19.15% (0.5%) ↓48.81% (1.5%) ↓42.55% (3%)	↑32.14% (0.5%) ↓15.63% (3%)		
				MDA thigh (1 d) thigh	NS	↑55.6% (0.5%) ↑66.7% (1.5%) ↑55.6% (3%)		
				MDA thigh (4 d) thigh	↓23.3% (3%)	↑52.6% (0.5%) ↑42.1% (1.5%) ↑21.1% (3%)		
				MDA thigh (7 d) thigh	↓13.95% (0.5%) ↓18.6% (1.5%) ↓30.23% (3%)	↑32.14% (0.5%) ↑25% (1.5%)		
			Hepatic vitamin E concentration	α-tocopherol in liver (21 d)	↑29.52% (1.5%) ↑33.3% (3%)	↓19.77% (0.5%)		
**Grape polyphenols**	0.0025 (LGPP),0.0050 (MGPP), 0075 (HGPP)	E, 100 mg/kg (Control), replaced with the ratio 0.0025, 0.0050, 0.0075 % of GPP	DPPH in breast and in legs	DPPH in breast		↓4.76% (LGPP) ↓14% (MGPP) ↓8.36% (HGPP)	35 d	Iqbal et al.,(2014)

				DPPH in leg	↓6.24% (LGPP) ↓13.75% (MGPP) ↓8.52% (HGPP)		
			Organ weights	Heart	↓29.2% (LGPP) ↓29.2% (MGPP) ↓32.82% (HGPP)		
				Liver	↓3.48% (LGPP)		
**Grape polyphenols**	0.0025, 0.0050, 0.0075	E, 100 mg/kg (Control),replaced with the ratio 25, 50, 75 mg/kg of GPP	Lipid oxidation	TPC in breast	↑9.39% (LGPP) ↑21.12% (MGPP) ↑30.61% (HGPP)	35 d	Iqbal et al., (2015)
				TPC in leg	↑8.35% (LGPP) ↑17.84% (MGPP) ↑26.49% (HGPP)		
				TBARS in breast	↓9.1% (LGPP) ↓27.3% (MPP) ↓18.2% (HGPP)		
				TBARS in leg	↓6.78% (LGPP) ↓23.73% (MPP) ↓19.5% (HGPP)		

**Grape pomace**	5, 7.5, 10	E, 200 mg/kg	Carcass traits, organ weights	Carcass, breast, thigh, liver, heart, abdominal fat	NS	NS	42 d	Ebrahimzadeh et al., (2018)
**Oregano plant (OP)**	3	E (200 mg/kg) + C (1000 mg/kg)	Lipid oxidation of chicken meat	TBARS in PM	NS	TN:160%; HS: ↑71.8%	42 d	Young et al., (2003)
				TBARS in IL	NS	HS: ↑88.3%		
				TBARS in liver	TN: ↓28.3% HS: ↓47.92%	NS		
			Meat color	L* in PM	NS	↓1.18%		
				b* in PM	NS	↑41,38%		
				b* in IL	↑56.7%	↑74.1%		
			Tocopherols concentration	α-Tocopherol in PM	NS	↓6.25%		
				γ-Tocopherol in PM	NS	NS		
**Oregano plant**	0.5,1, 0.5+ 170 mg/kg of VitE (OR5E), 1+ 170 mg/kg of VitE (OR10E)	E, Flavomicin + lasalocid group (positive control)	Lipid oxidation of chicken meat	MDA	↓ (0.5%, 1%, OR5E, OR10E)	↑ (0.5%, 1%)	42 d	Giannenas et al., 2005
**Oregano essential oil (turkeys)**	0.01, 0.02, 0.01 + 100 mg/kg of VitE	E, 200 mg/kg	Lipid oxidation in refrigerated storage	MDA in refrigerated storage BM (0 d)	↓17.1% (0.01%) ↓32.49% (0.02%) ↓39.38% (OOE)	↑22.85% (0.01%); =0.02%=OOE	28 d	Botsoglou et al., (2003b)

				MDA in refrigerated storage TM (0 d)	↓22% (0.01%) ↓36.48% (0.02%) ↓41.35% (OOE)	↑24.62% (0.01%); =0.02%=OOE		
				MDA in refrigerated storage in BM and in TG after 3, 6, 9 days	↓ OO (0.01%)>OO (0.02%)	↑24.62% (0.01%); =0.02%=OOE		
**Oregano (OO) essential oil (turkeys)**	0.01, 0.02, 0.01 + 100 mg/kg of VitE (OOE)	E, 200 mg/kg	Lipid of turkey meat in long term frozen	MDA in frozen storage after 3 months in BM	↓ OO (0.01%)>OO (0.02%)	=0.0200=OOE	28 d	Botsoglou et al., (2003a)
				MDA 6 months BM	↓ 0.0100>0.0200	=0.0200=OOE		
				MDA 9 months BM	↓ 0.0100>0.0200	=0.0200=OOE		
				MDA after 7 d of refrigerated display (following 9 months of frozen)	↓ 0.0100>0.0200	=0.0200>OOE		
				α-tocopherol in BM (0 months)	↑ 20.95% (0.01%) ↑36.2% (0.02%) ↑71.43% (OOE)	↓42.27% (0.01%) ↓35% (0.02%) ↓18.2% (OOE)		

				α-tocopherol in BM (9 months)	↑ 42.62% (0.01%) ↑72.13% (0.02%) ↑122.95% (OOE)	↓32% (0.01%) ↓17.97% (0.02%)		
				α-tocopherol TG (0 months)	↑ 18.85% (0.01%) ↑40.98% (0.02%) ↑190.98% (OOE)	↓32% (0.01%) ↓17.97% (0.02%)		
				α-tocopherol TG (9 months)	↑ 24.29% (0.01%) ↑60% (0.02%) ↑291.4% (OOE)	↓79.86% (0.01%) ↓74.1% (0.02%) ↓36.57%		
**Oregano essential oil (broilers)**	0.005%, 0.01%	E, 200 mg/kg	Lipid oxidation	MDA in BM stored at -20 °C 3 months	↑23.22% (0.005%) ↓38.76% (0.01%)	↑60.78% (0.005%) ↑28.24% (0.01%)	38 d	Botsoglou et al., (2003)
				6 months	↓19.2% (0.005%) ↓39.1% (0.01%)	↑95.2% (0.005%) ↑47.2% (0.01%)		
				9 months	↓15.38% (0.005%) ↓41.23% (0.01%)	↑50.46% (0.005%) ↑50.46% (0.01%)		
**Oregano oil (OO)**	0.05%, 0.01%	E, 200 mg/kg	Lipid oxidation of chicken meat	MDA on iron induced lipid oxidation in BM after 150 min	↓0.05>0.1	↑0.05>0.1	38 d	Botsoglou et al., (2002)

				MDA on iron induced lipid oxidation in TM after 50 min	↓ (0.1%)	↑ (0.1%)		
				MDA on iron induced lipid oxidation in TM after 100 and 150 min	↓ 0.5>0.1	↑ (0.1%)		
**Oregano plant (OP)**	0.5% (OP); 0.5% +190 mg/kg of VitC (OC), or + 170 mg/kg (OE), or with both (OCE)	Vitamin C (VitC), Vitamin E (VitE), Vit C+ VitE (CE)	Lipid oxidation during refrigerated storage	MDA in raw BM, TM (across 9 days)	↓ OP=OC>OE=OCE	↑ OP=OC>OE=OCE		Florou-Paneri et al. (2006)
				MDA in cooked BM, TM	↓ OP (0.5%)	↓OC=OCE; ↑O=OC		
**Oregano oil (CSB=crude soybean oil diet; ASO = acidulated soybean oil soapstock)**	0.01	E, 10 mg/kg (E10), 100 mg/kg (E100)	FA profile of BM in both diets	From C14:0 to C18:2,C20:4,SFA,MUFA,PUFA,PUFA:SFA		NS	42	Avila-Ramos et al. (2012)
				C18:3 (CSB)		E10:↑ 38.43% E100: ↑44.44%		
				C18:3 (ASO)		NS		

			Lipid oxidation	MDA in cooked meat strips, 9 d of storage (CSB)		↓E10=E100		
				MDA in cooked meat strips, 9 d of storage (ASO)		↑E10>E100		
				MDA in cooked meat patties, after 6 d of storage (ASO)		↓E10 ↑E100		
				MDA in cooked meat patties, after 6 d of storage (ASO)		↑E10>E100		
**Oregano oil**	0.01, 0.02, 0.01+100 mg/kg of VitE (OOE)	E, 200 mg/kg	Iron-induced lipid oxidation in chicken meat, liver and heart	MDA prior iron induced lipid oxidation in all tissues	↓ 0.0200=OOE $P<0.05$	↑0.0100 $P<0.05$	28 d	Papageorgiou et al. (2003)
				MDA after iron induced lipid oxidation in BM at all points	↓ 0.0100 >0.0200>OOE	↑0.0100 $P<0.05$		

	MDA after iron induced lipid oxidation in BM after 150 min	↓ 0.0100 >0.0200>OOE	= 0.0200; ↓OOE
	MDA after iron induced lipid oxidation in TM after 150 min	↓ 0.0100 >0.0200>OOE	= 0.0200; ↓OOE
	MDA after iron induced lipid oxidation in heart and liver	similar trend	similar trend
Tocopherol concentration in differen tissue	BM	↑15.24% (0.01%) ↑30.48% (0.02%) ↑71.43% (OOE)	↓45% (0.01%) ↓37.73% (0.02%) ↓18.1% (OOE)
	TM	↑14.75% (0.01%) ↑38.52% (0.02%) ↑190.98% (OOE)	↓75.95% (0.01%) ↓71.1% (0.02%) ↓39% (OOE)
	Liver	↑39.9% (OOE)	↓42.12% (0.01%) ↓40.56% (0.02%) ↓21.06% (OOE)

				Heart	↑17.86% (0.01%) ↑50.71% (0.02%) ↑487.1% (OOE)	↓86.48% (0.01%) ↓82.7% (0.02%) ↓32.62% (OOE)		
**Tomato pomace**	30	E, 242 mg/Kg	Lipid oxidation	TBARS after 4 days of storage	↓23%		21 d	King and Zeidler, (2004)
**Commercial essential oils**	0.05, 0.1	E, 200 mg/kg	Lipid oxidation: MDA in thigh (TM) and breast muscle (BM) (both raw and heat treated (HT) tissue, Vitamin E	MDA TM (Raw tissue)	↓27.41% (0.05%) ↓47.9% (0.1%)	↑83.14% (0.05%)	42 d	Botsoglou et al. (2004)
				MDA TM (HT tissue)	↓34.38% (0.05%) ↓56.17% (0.1%)	↑263.2% (0.05%) ↑142.6% (0.1%)		
				MDA BM (Raw tissue)	↓22.4% (0.05%) ↓38.3% (0.1%)	↑53.41% (0.05%)		
				MDA BM (HT tissue)	↓36.42% (0.05%) ↓62.3% (0.1%)	↑136.6% (0.05%) ↑40.3% (0.1%)		
				α-tocopherol TM (Raw tissue)	NS	↓79.27% (0.05%) ↓79.63%(0.1%)		
				α-tocopherol TM (HT tissue)	NS	↓81.59% (0.05%) ↓80.9%(0.1%)		

				α-tocopherol BM (Raw tissue)	NS	↓77.85% (0.05%) ↓77.17%(0.1%)		
				α-tocopherol BM (HT tissue)	NS	↓80.69% (0.05%) ↓78.74% (0.1%)		
**Rosemary powder (RP)**	0.5, 1.0 , 0.5 or 1+100 mg/kg of VitE (0.5RPE, 1RPE), or + 200mg/kg of VitE (0.5RPE2, 1RPE2)	E, 200 mg/kg (VitE2)	Lipid oxidation in BM stored at 4°C for 14 days	MDA d 4	↓21.82% (0.5RPE), ↓23.64% (0.5RPE2) ↓25.45% (1RPE2)	NS	42 d	Rostami et al. (2017)
				MDA d 7	↓11.94% (0.5RPE), ↓10.45% (0.5RPE2) ↓11.94% (1RPE2)	NS		
				MDA d 14	↓20.73% (0.5%), ↓23.17% (0.5RPE) ↓26.83% (0.5RPE2); ↓19.5% (1%) ↓24.39% (1RPE2)	NS		

			Carcass traits	Right cecum weight, right cecum length, left cecum length; Full abdomen carcass weight, empty abdomen carcass weight, eviscerated carcass, breast weight, relative breast weight, drumsticks weight, relative drumsticks weight	NS	NS		
				Relative weight of right cecum	NS	VitE2: ↑85.71% (0.5%)		
**Olive leaves (turkey)**	0.5, 1	E, 150 mg/kg (E150), 300 mg/kg (E300)	Lipid oxidation of turkey meat during refrigerated storage	TBARS across the entire storage period	↓ 0.5OL>1OL $P<0.05$	E150: ↓1OL E300: ↑0.5OL>1OL $P<0.05$	42 d	Botsoglou et al. (2010)

			Bacteria count as meat: TVC (Total viable counts, LAB (Lactic acid bacteria), ENB (Enterobacteriaceae), PSY	Bacterial count at day 2 and afterwards	↓ 0.5OL=1OL P <0.05	↓ 0.5OL=1OL P <0.05		
				Bacterial count at day 2 and afterwards (TVC, LAB, ENB, PSY)	↓ 0.5OL<1OL P <0.06	↓ 0.5OL<1OL P <0.06		
				TVC count at day 12 of storage (range)	↓19.74% (0.5%) ↓32.9% (1%)	↓19.74% (0.5%) ↓32.9% (1%) to both groups of VitE		
				LAB count at day 12 of storage (range)	↓28.47% (0.5%) ↓40.15% (1%)	↓28.47% (0.5%) ↓40.15% (1%)		
				ENB count at day 12 of storage (range)	↓21.62% (0.5%) ↓36.49% (1%)	↓21.62% (0.5%) ↓57.26% (1%)		
				PSY count at day 12 of storage (range)	↓21.62% (0.5%) ↓36.48% (1%)	↓21.62% (0.5%) ↓36.48% (1%)		
**Rosemary plant (RP), rosemary volatile oil (RO)**	R: 0.57, 0.86, 1.15; RO 0.01, 0.015, 0.02	E, 200 mg/kg	Lipid oxidation (average of the 3 groups)	MDA d 1		↓25.7%% (RP) ↓28.57% (RO)	42 d	Yesilbag et al. (2011)

				MDA d 3		↓26.32%(RP) ↓24.56% (RO)		
				MDA d 5		↓29.87%(RP) ↓23.38% (RO)		
			Bacteria counts on meat	*E. coli*		↑75.95%(RP) ↑56.96% (RO)		
			Meat pH	BM		↓1.19% (RP)		
				TM		↓0.84% (RP)		
**Rosemary leaves (RL)**	0.5, 1	E, 300 mg/kg	Lipid oxidation of turkey meat	MDA in raw BM stored at 4°C for up to 12 days	↓ 0.5RL>1RL $P < 0.05$	↑0.5RL>1RL P <0.05	28 d	Govaris et al. (2007)
			Microbial growth	Bacterial count d 2	↓ 0.5RL>1RL P <0.05			
				TVC d 12	↓ 11.1% (0.5%) ↓ 24.2% (1%)	↓ 11.1% (0.5%) ↓ 24.2% (1%)		
				PSY d 12	↓12.93% (0.5%) ↓23.81% (1%)	↓12.93% (0.5%) ↓23.81% (1%)		
				LAB d 12	↓11.63% (0.5%) ↓24% (1%)	↓11.63% (0.5%) ↓24% (1%)		
				ENB d 12	↓15% (0.5%) ↓25.7% (1%)	↓15% (0.5%) ↓25.7% (1%)		

**Rosemary leaves and olive leaves (OL)**	1	E, 150 mg/kg (E150), 300 mg/kg (E300)	Lipid oxidation of turkey meat during refrigerated storage	MDA in raw BM stored at 4°C for up to 12 days (all time points)	↓RL>OL P <0.05	E150: ↓RL>OL P<0.05: E300: ↑RL>OL P<0.05	42 d	Govaris et al. (2010)
			Microbial growth	TVC d 12	↓16.13% (RL) ↓32.9% (OL)	↓16.13% (RL) ↓32.9% (OL)		
				PSY d 12	↓17.57% (RL) ↓40.54% (OL)	↓17.57% (RL) ↓40.54% (OL)		
				LAB d 12	↓15.33% (RL) ↓38.69% (OL)	↓15.33% (RL) ↓38.69% (OL)		
				ENB d 12	↓21.37% (RL) ↓40.17% (OL)	↓21.37% (RL) ↓40.17% (OL)		
**Rosemary leaves (RL)**	0.5 (0.5RL), 1 (1RL), 0.5 (0.5RLE) or 1 (1RLE)+300 mg/kg of VitE	E, from 10 to 300 mg/kg	Lipid oxidation	MDA in raw meat samples	↓>0.5RL>1RL>0.5RLE=1RLE	↑>0.5RL>1RL>0.5RLE=1RLE	16 weeks	Botsoglou et al. (2007)
				α-tocopherol in BM	↑185.7% (0.5RLE) ↑177.4% (1RLE)	↓59.52% (0.5RL) ↓61% (1RL)		
				α-tocopherol in TM	↑453% (0.5RLE) ↑442.6% (1RLE)	↓33.54% (0.5RL) ↓79.81% (1RL)		

**Plant extract: Rosemary (RO), Green tea (GT), Grape seed (GS), Tomato extracts (TO)**	0.01, 0.02 each, other combinations	E, 200 mg/kg along with 300 mg/kg of synthetic antioxidants, 300 mg/kg of synthetic antioxidants (SYNT)	Fatty acid profile (FAP); Lipid oxidation, Protein oxidation; α-tocopherol content in muscle	TBARS		↑ all	42 d	Smet et al. (2008)
**Oregano (OO) and rosemary (RO) essential oils**	0.015OO: 0.015%; 0.03OO: 0.03%; 0.015RO: 0.015%; 0.03RO: 0.03%; 0.0075OO+0.0075RO: both 0.0075%; 0.015OO150+0.015RO: 0.015%	E, 200 mg/kg	Lipid oxidation: MDA in stored meat enriched with n-3 PUFAs	MDA in BM (d 0)	from ↓67.26% (0.015OO) to ↓85.84% (0.015OO+0.015RO)	↑ 60.87% (0.015OO) ↑34.78% (0.03OO) ↑26.1% (0.03RO)	21 d	Basmacioglu et al., (2004)
				MDA in BM (d 15)	from ↓85.3% (0.015OO) to ↓93.96% (0.015OO+0.015RO)	↑ 17.74% (0.015OO) ↓37.1 % (0.075OO+0.075RO) ↓51.6% (0.015OO-0.015RO)		
				MDA in TM (d 0)	from ↓ 64.8% (0.015OO) to ↓84% (0.015OO+0.015RO)	↑ 51.72% (0.015OO) ↑ 31% (0.03OO) ↓31.03% (0.015OO+0.015RO)		

MDA in TM (d 15)	from ↓84.97% (0.015OO) to ↓93.79% (0.015OO+0.015RO)	↓10.45% (0.015RO) ↓17.91% (0.03RO) ↓38.81% (0.075OO+0.075RO) ↓53.73% (0.015OO+0.015RO)
α-tocopherol BM (d o)	↑ 8% (0.03RO)	↓ from 80.99% (0.03RO) to 83.1% (0.015OO)
α-tocopherol BM (d 15)	↑ 400% (0.015RO) ↑ 500% (0.03RO) ↑ 600% (0.075OO+0.075RO) ↑ 700% (0.015OO+0.015RO)	↓ from 99.2% (0.015OO and 0.015OO) to 93.94% (0.015OO+ 0.015RO)
α-tocopherol TM (d 0)	↑10% (except 0.015RO)	↓ from 92.57% (except 0.015RO) to 93.24% (0.015OO+ 0.015RO)
α-tocopherol TM (d 15)	↑400% (0.03RO, 0.075OO+0.075RO) ↑600% (0.015OO+0.015RO)	↓ from 99.1% (0.015OO, 0.03OO) to 93.58% (0.015OO+0.15RO)

**Rosemary leaves (RL), rosehip fruits (RF), chokeberry pomace (CP), entire nettle (EN)**	2.5 of each alternative	E, 200 mg/kg	Slaughter traits (carcass weight, dressing %, organg/kg carcass)	Pancreas weight	↑ 18.2% (RL)	↑ 21.46% (RL)	28 d	Loetscher et al. (2013)
				Liver weight	↑ 27.56% (RL) ↑15.1% (EN)	↑ 18.6% (RL)		
			Color of skin,breast, liver	Skin color yellowness	↑29.63% (RL) ↑90.74% (EN)	↑77.59% (EN)		
				Liver color b*	↑33.69% (EN)	↑26.83% (EN)		
			Tocopherols in breast meat	α	↑68.13% (RL) ↑92.8% (EN)	↓57.4% (RL) ↓67.85% (RF) ↓69.6% (CP) ↓51.15% (EN)		
				γ	NS	↓44.91% (CP)		
				Total	↑62% (RL) ↑84.42% (EN)	↓56.44% (RL) ↓66.1% (RF) ↓68.5% (CP) ↓50.42% (EN)		

Lipid oxidation of meat: breast meat tocopherols, TBARS, resistance against oxidation (induction time) of the abdominal fat	TBARS in breast meat (d 0)	↓6.65% (RF) ↓29.3% (CP)	NS
	TBARS in breast meat (d 3)	NS	NS
	TBARS in breast meat (d 6)	NS	↑496.6% (RF) ↑909.2% (EN)
	TBARS in breast meat (d 9)	↓57.19% (RL)	↑335.6% (RF) ↑370.53% (CP) ↑590.86% (EN)
	Resistance against oxidation of the abdominal fat	↑32.62% (RL)	↓ 42.46% (RL) ↓54.1% (RF) ↓49.65% (CP) ↓55.7% (EN)

**Hesperidin (HE)**	0.15, 0.3	E, 200 mg/kg	Cold CW, internal organ weights, pH24, Shear values, intramuscular fat, cooking loss, color. Lipid oxidation (MDA), Meat pH	Cold CW	NS	NS	40 d	Simitzis et al. (2011)
				pH24	NS	↓0.99% (0.3%)		
				Color L*	NS	↑4.48% (0.15%)		
				Color a*	NS	↓15.9% (0.15%)		
				Color b*	NS	↓8.63(0.15%) ↓6.36% (0.3%)		
				MDA (1 d)	NS	↑109.8% (0.15%) ↑77.99% (0.3%)		
				MDA (3d)	↓35.37% (0.3%)	↑215.2% (0.15%) ↑139.7% (0.3%)		
				MDA (6 d)	↓31.2% (0.15%) ↓40% (0.3%)	↑399% (0.15%) ↑335.2% (0.3%)		
				MDA (9d)	↓37.8%(0.15%) ↓46.5% (0.3%)	↑361.3% (0.15%) ↑299.6% (0.3%)		

**Coneflower (CE), Thyme (TE), Sage (SE), Marigold extracts (ME)**	0.056%, 0.002% ME	E; BHT+EQ+BHA, (SYNT1); xantophyll, (SYNT2	Slaughter traits; Selected fatty acids of BM	C18:0	↑20.1% (SE),	NS	20 d	Koreleski and Swiatkiewwicz (2007)
				C18:1	↓12.75% (SE)	SYNT2: ↓11.56% (SE)		
				C18:3	↑550% (SE)	SYNT2: ↓24.55% (SE)		
				C20:4	↑44.72% (SE)	SYNT1: 31.19%; SYNT2: ↑60% (SE)		
				C22:6 n-3 (DHA)	↑59.58% (SE)	SYNT2: ↑%(SE)		
				PUFA n-3	NS	SYNT2: ↑33.97%(SE)		
				n-6/ n-3	NS	SYNT1: ↓24.46% SYNT2: ↓23.44%(SE)		
			Lipid oxidation	TBARS	↑36.48% (ME)	ViTE: ↑33.68% (ME)		
				Vitamin E	NS	VitE: ↓72.1% (CE) ↓70.7% (TE) ↓68.7% (ME)		

			Sensory properties	Flavour	NS	SYNT2:↑10.34% (CE) ↑9.61% (TE) ↑7.64% (ME)		
				Taste	NS	SYNT2: ↑9.5% (CE) ↑13% (TE) ↑11.75% (SE)		
**Tea catechins (TC)**	0.005, 0.01, 0.02, 0.03	E, 200 mg/kg	Lipid oxidation	TBARS in BM after incubation of 100 min	↓ 0.02TC=0.03TC; ↑0.005TC P<0.05		42 d	Tang et al., (2000)
				TBARS in BM after incubation of 200 min	↓ 0.02TC=0.03TC; ↑0.005TC P<0.06	↓ 0.02TC=0.03TC		
				TBARS in TM after incubation of 50 min and 100 min in TM	↓ 0.02TC=0.03TC=0.01TC P<0.05			
				TBARS in TM after incubation of 200 min	↓0.02TC=0.03TC P<0.05	↓0.03TC		
				TBARS in liver up to 200 min	↓0.03TC P<0.05			
				TBARS in liver 200 min				

**Tea catechins**	0.005, 0.01, 0.02, 0.04	E, 200 mg/kg	Iron-induced lipid oxidation in chicken meat, liver and heart stored at -20° C	TBARS in BM (3 months), similar trend afterwards	↓62.79% (0.02%)	↓9.1% (0.03%) P<0.05; ↑300% (0.005%) ↑181.(& (0.01%) ↑45.% (0.02%) P<0.001	42 d	Tang et al., (2001)
				TBARS in TM (3 months)	Similar results	Similar reuslts		
**Tomato skin (TS), orange peel (OE), green tea leaves (GTL) (broilers)**	200 mg/kg each	E, 200 mg/kg	Slaughter traits, meat traits, Lipid oxidation	TBARS (average) in BM	↑39.13% (TS) ↑19.57% (OE)	↑56.1% (TS) ↑34.2% (OE)	56 d	Marzoni et al. (2014)
				TBARS (average) in TM	↓55.18% (TS) ↓62.44%(OE) ↑24.87% (GTL)	↑212.99% (GTL)		
**Rosemary leaves (RL) , orange peel (OP)(ducks)**	200 mg/kg each	E, 200 mg/kg	Slaughter traits, meat traits, Lipid oxidation	TBARS (average) in BM	↓46.34% (OE) ↓46.78% (RL)	↑287.5% (OE) ↑95.83% (RL)	69 d	Marzoni et al. (2014)
				TBARS (average) in TM	↓33.1% (OE) ↓66.2% (RL)	↑86.2% (OE) ↑84.62% (RL)		
**Sweet chestnut wood extract (SCW)**	0.3% or with VitE (SCWE), CP: diet, CLIN	E, 85 mg/kg (85VitE), 200 mg/kg (200VitE)	Fatty acid composition of BM	Total fat	CP: ↑78.49% (0.3%), ↑97.85% (SCWE); CLIN: NS	NS	25 d	Voljc et al. (2013)

C18:1 n-9	CP: ↑39.21% (0.3%), ↑45% (SCWE); CLIN: NS	NS
C18:2 n-6	CP: ↑104.8% (0.3%), ↑129% (SCWE); CLIN: NS	NS
C20:4 n-6	CP: ↓30.65% (0.3%), ↓31.5% (SCWE); CLIN: NS	NS
C20:5 n-3	CP: ↑561.7% (0.3%), ↑574.65% (SCWE); CLIN: ↑19.91% (0.3%) ↑22.27% (SCWE)	85VitE: ↑8.3% (0.3%) ↑10.42% (SCWE); 200VitE: ↑13.2% (0.3%) ↑15.42% (SCWE)
C22:6 n-3	CP: ↑54.23% (0.3%), ↑56% (SCWE); CLIN: NS	NS
n-3 PUFA	CP: ↑1089.5% (0.3%), ↑1399.76% (SCWE); CLIN: NS	NS

				n-6 PUFA	CP: ↑74.6%(SCWE)	NS	
				n-6/n-3	CP:↓89.1% (0.3%) ↓89.1% (SCWE)		
			Meat quality characteristics	Lightness d 7	NS	85VitE: ↑7.11% (SCWE)	
				Yellowness (b*) d 1	CP: ↑109.9% (SCWE); CLIN: ↑49.5&% (SCWE)	85 VitE: ↑50.2% (SCWE)	
				Yellowness (b*) d 7	CP: ↑31% (SCWE)	85VitE: ↑19.65% (SCWE)	
			Lipid oxidation	MDA in raw BM	CP: ↑505.3% (SCWE)	85 VitE: ↑225% (SCWE)	
				MDA in heat stress BM	CP: ↑172.69% (SCWE); CLIN: ↓47.3% (SCWE)	85 VitE: ↑50.1% (0.3%) 200VitE ↑154.4% (0.3%)	
**Polyphenol product (PP)**	0.01%+100 mg (PPE) of VitE;0.02% (PP)	E, 100 mg/kg (100VE), 200 mg/kg (200VE)	Carcass quality , structure gastrointestinal tract, digesta in pH	BM content of carcass (%)	HS: ↑7.4% (PPE)	100VE: ↑6.63% (PPE)	Mazur-Kusnirek et al., 2019a
			Meat quality characteristics	Colour, b*	TN: ↓11.76% (PPE) ↓13.18% (PP)	NS	

	Natural drip loss	HS: ↓39.87% (PPE) ↓37.34% (PP)	
	WHC	HS: ↓28.71% (PPE) ↓24.95% (PP)	NS
	Crude ash	TN: ↑9.82% (PP) HS:↑6%(PP)	NS
FAC of BM	PUFA	HS: ↑7.7% (PPE) ↑8.62% (PP)	NS
	n-6	HS: ↑6.54% (PPE) ↑7% (PP)	NS
	C18:1 n-9	TN:↑41.2%; HS:↓2.84% (PP)	NS
	C20:2	HS: ↑27.78% (PPE) ↑22.2% (PP)	NS
	C20:4	HS: ↑53.9% (PPE) ↑51.56% (PP)	NS
	C20:5	HS: ↑93.3% (PP)	NS

				C22:0	HS: ↑34.48% (PPE), HS: ↑34.48% (PP)	NS		
				C22:5	HS: ↑40.82% (PPE), HS: ↑40.82% (PP)	NS		
**Oregano aqueous extract (OaE)**	0.02	E, 150 mg/kg	Meat quality characteristics of breast muscle	pH45', pH24	NS	NS	42	Forte et al. (2018)
				Color parameters	NS	NS		
				Drip, cooking loss, shear force	NS	NS		
				Moisture, protein, fat, ashes	NS	NS		
			Lipid oxidation	TPC	↑26.67%	↑32.56%		
				$ORAC_{FL}$	↑19.42%	NS		
			FAC of BM	NS	NS	NS		
			CLA isomers and PUFA n-3 content	NS	NS	NS		
			Attributes of meat (blind)	Appearance, texture, taste, overall liking	NS	NS		
			Attributes of meat (informed)	Appearance	↑16.55%	↑7.55%		

				Texture	↑16.43%	NS		
				Taste	↑22.12%	↑13.41%		
				Overall liking	↑23.11%	↑11.08%		
			Attributes of meat (expected)	Overall liking (only evaluated)	↑20%	↑7.53%		
**Sage extract (SE)**	2.5	E, 30 mg/kg	Slaughter traits	CW, CY	NS	NS	28	Loetscher et al. (2014)
				Sausage meat yield (/CW or /CY)	NS	NS		
			Lipid oxidation of abdominal fat and sausages	Induction time (IT)	NS	NS		
				TBARS in raw sausage material (1, 4, 10 mon)	NS	NS		
				TBARS in raw sausage material (7 mon)	↓21.4%	NS		
				TBARS in spiced sausages (1, 4,7 10 mon)	NS	NS		
			Microbial counts	Raw sausage material (1, 10 mon)	NS	NS		
				Spiced sausages (1, 10 mon)	NS	NS		

**Thyme oil**	0.01, 0.02	E, 100 mg/kg (E100); 200 mg/kg (E200)	Carcass traits, organ weights	Hot CW	↓4.08% (0.01%)	E100:↑3.85% (0.02%) E200: ↓4.66% (0.01%)	42	Bölükbaşi et al. (2006)
				Liver weight	↓6.98% (0.01%) ↓4.65% (0.02%)	E100: NS E200: ↓13.04% (0.01%) ↓10.87% (0.02%)		
				Heart, breast, leg	NS	NS		
				Wing	↓8.16% (0.01%)	E100: ↓2.17% (0.01%) ↑1.63 % (0.02%) E200: ↓6.74% (0.01%)		
				Hot CY	↓2.6% (0.01%)	E100: ↑1.32% (0.02%) E200: ↓3.23% (0.01%)		
			Lipid oxidation	TBARS in leg (d 1)	↓75.76% (0.01%) ↓88.9% (0.02%)	E100:↑33.33% (0.01%) ↓38.89% (0.02%) E200: ↑50% (0.01%) ↓31.25% (0.02%)		

TBARS in leg (d 3)	↓77% (0.01%) ↓88% (0.02%)	E100:↑64.29% (0.01%) ↓14.29% (0.02%) E200: ↑35.3% (0.01%) ↓29.41% (0.02%)
TBARS in leg (d 7)	↓95.43% (0.01%) ↓97.51% (0.02%)	E100:↑46.67% (0.01%) ↓20% (0.02%) E200: ↑22.2% (0.01%) ↓33.3% (0.02%)
TBARS in BM (d 1)	↓77.03% (0.01%) ↓86.49% (0.02%)	E100:↑88.89% (0.01%) ↑ 11.11% (0.02%) E200:E100:↑88.89% (0.01%) ↑ 11.11% (0.02%)
TBARS in BM (d 3)	↓77.63% (0.01%) ↓85.53% (0.02%)	E100:↓35.29% (0.02%) E200: ↑88.89% (0.01%) ↑22.22% (0.02%)
TBARS in BM (d 7)	↓96.11% (0.01%) ↓97.41% (0.02%)	E100:↓25% (0.01%) ↓50% (0.02%) E200: ↑50% (0.01%)

FA profile in leg	C14:0	↓3.59% (0.01%) ↓4.79% (0.02%)	E100:↓3.01% (0.01%) ↓4.22% (0.02%) E200:↓3.59% (0.01%) ↓4.79% (0.02%)
	C16:0	NS	NS
	C16:1	↑59.05% (0.01%) ↑138.36% (0.02%)	E100: ↑60.44% (0.01%) ↑140.44% (0.02%) E200: ↑59.74% (0.01%) ↑139.39% (0.02%)
	C18:0	↓10.37% (0.01%) ↓32.68% (0.02%)	E100: ↓10.37% (0.01%) ↓32.68% (0.02%) E200: ↓10.2% (0.01%) ↓32.55% (0.02%)
	C18:1 cis-9	↓5.33% (0.02%)	E100: ↓5.4% E200: ↓5.33% (0.02%)

C18:2	↑8.77% (0.01%) ↑11.5% (0.02%)	E100: ↑8.77% (0.01%) ↑11.5% (0.02%) E200: ↑8.81% (0.01%) ↑11.53% (0.02%)
C18:2n-6	↓57.14% (0.01%) ↓61.06% (0.02%)	E100: ↓57.14% (0.01%) ↓61.06% (0.02%) E200: ↓57.14% (0.01%) ↓61.06% (0.02%)
C20:4, ω-6	↓51.1% (0.01%) ↓48.35% (0.02%)	E100: ↓50.93% (0.01%) ↓48.16% (0.02%) E200: ↓51.01% (0.01%) ↓48.25% (0.02%)
SFA	↓2.53% (0.01%) ↓6.92% (0.02%)	E100: ↓2.45% (0.01%) ↓6.85% (0.02%) E200: ↓2.49% (0.01%) ↓6.89% (0.02%)

	MUFA	↑4.59% (0.01%) ↑6.1% (0.02%)	E100: ↑4.59% (0.01%) ↑6.1% (0.02%) E200:↑4.63% (0.01%) ↑6.14% (0.02%)
	PUFA	↓4.35% (0.01%) ↓2.18% (0.02%)	E100: ↓4.31% (0.01%) ↓2.13% (0.02%) E200: ↓4.31% (0.01%) ↓2.13% (0.02%)
	UP	NS	NS
FA profile in BM	C14:0	↓21.64% (0.01%) ↓34.21% (0.02%)	E100: ↓21.41% (0.01%) ↓34.02% (0.02%) E200: ↓21.87% (0.01%) ↓34.40% (0.02%)
	C16:0	↑4.3% (0.01%) ↑4.3% (0.02%)	E100: ↑6.25% (0.01%) ↑6.25% (0.02%) E200:↑6.25% (0.01%) ↑6.25% (0.02%)

C16:1	↑51.36% (0.01%) ↑131.36% (0.02%)	E100: ↑51.36% (0.01%) ↑131.36% (0.02%) E200:↑50.68% (0.01%) ↑130.32% (0.02%)
C18:0	↓6.54% (0.01%) ↓41.52% (0.02%)	E100: ↓6.21% (0.01%) ↓4.31% (0.02%) E200: ↓6.21% (0.01%) ↓4.31% (0.02%)
C18:1 cis-9	↑3.53% (0.01%) ↑6.57% (0.02%)	E100: ↑3.57% (0.01%) ↑6.61% (0.02%) E200: ↑3.53% (0.01%) ↑2.93% (0.02%)
C18:2	↑2.94% (0.01%) ↑9.40% (0.02%)	E100: ↑2.91% (0.01%) ↑9.37% (0.02%) E200: ↑2.94% (0.01%) ↑9.40% (0.02%)

C18:2n-6	↓15.86% (0.01%) ↓12.41% (0.02%)	E100: ↓15.86% (0.01%) ↓12.41% (0.02%) E200: ↓15.28% (0.01%) ↓11.81% (0.02%)
C20:4, ω-6	↓27.72% (0.01%) ↓49.75% (0.02%)	E100: ↓27.54% (0.01%) ↓49.63% (0.02%) E200: ↓27.45% (0.01%) ↓49.57% (0.02%)
SFA	↓0.68% (0.01%) ↓10.04% (0.02%)	E100: ↓0.44% (0.01%) ↓9.82% (0.02%) E200: ↓0.36% (0.01%) ↓9.42% (0.02%)
MUFA	↑5.47% (0.01%) ↑16.19% (0.02%)	E100: ↑5.5% (0.01%) ↑16.23% (0.02%) E200: ↑5.43% (0.01%) ↑16.15% (0.02%)

				PUFA	↓3.75% (0.01%) ↓2.99% (0.02%)	E100: ↓3.72% (0.01%) ↓2.97% (0.02%) E200: ↓3.65% (0.01%) ↓2.9% (0.02%)	
				UP	NS	NS	
**Polyphenols product**	0.01+100 mg/kg of VitE (0.01E), 0.02,	E, 100 mg/kg (E100), 200 mg/kg, (E200)<br><br>2 control, 1 negative (without low quality oil), 1 positive (with low quality oil)	Antioxidant status in BM	Vitamin C	NS	NS	Mazur-Kusnirek et al. (2019b)
				Retinol	NO: ↓18.75% (0.02%) WO: ↑41.67% (0.01E)	E100: ↓23.53% (0.02%) E200: ↓18.75% (0.02%)	
				Total tocopherols	NO: ↑287.1% (0.01E) =0.02% WO: ↑346.91% (0.01E) =0.02%	E100: ↓23.53% (0.02%) E200: ↓37.4% (0.01E) ↓84.19% (0.02%)	

	Total tocopherols	NO: ↑287.1% (0.01E) =0.02% WO: ↑346.91% (0.01E) =0.02%	E100: ↓23.53% (0.02%) E200: ↓37.4% (0.01E) ↓84.19% (0.02%)
	Vitamin EEq	NO: ↑355.1% (0.01E) =0.02% WO: ↑383.52% (0.01E) =0.02%	E100: ↑15.47% (0.01E) ↓73.41%% (0.02%) E200: ↓37.38% (0.01%) ↓85.58% (0.02%)
	TBARS	WO: ↓82.67% (0.01E)	NS
Carcasse traits, organ weights, pH digesta	Carcass dressing %	NS	E100: ↑3.42% (0.02%) P=0.064
	BM content of carcass, Heart content of BW, Liver content of BW, Abdominal fat content of BW, Crop weight and digesta pH, Proventriculus weight and digesta pH	NS	NS

	Guizzard weight	NS	E100: ↓15.61%
	Digesta pH	NS	NS
	Small intestine and ceca traits	NS	NS
Physicochemical properties of BM	pH15	NO: ↑5.05% (0.01E) ↑3.75% (0.02%) WO: NS	NS
	pH24	NS	E200: ↓3.81% (0.01E) ↓2.65% (0.02%)
	Colour		
	L*	NO: ↑3.45% (0.01E) ↑4.97% (0.02%) WO: ↑3.66% (0.01E) ↑5.18% (0.02%)	E100: ↑4.13% (0.2%) E200: ↑3.84% (0.01E) ↑5.37% (0.02%)
	a	NS	NS
	b	NS	NS
	Natural drip loss, WHC	NS	NS
	Dry matter, crude protein	NS	NS
	Crude fat	NO: ↑54.05% (0.02%)	NS

	Crude ash	NO: ↑8.04% (0.01E) ↑9.82% (0.02%) WO: NS	NS
FAC of BM	SFAs	NS	NS
	MUFAs	NO:↑6.1% (0.01E) ↑8.77% (0.02%)	NS
	PUFAs	NO: ↓17.64% (0.02%) WO: ↑10.14% (0.01E)	NS
	n-3, n-6/n-3	NS	NS
	DFAs	NO: ↓2.04% (0.02%) WO: ↑1.46% (0.01E)	E100: ↓1.54% (0.02%) E200: ↓1.71% (0.02%)
	OFAs	WO: ↓5.21% (0.01E)	E100: ↑6.11% (0.02%) E200: ↑6.85% (0.02%)
	DFA/OFA	NO:↓9.56% (0.02%) WO: NS	NS
	AI	NO: ↑10.71% (0.02%) WO: ↓6.25% (0.01E)	E100: ↑6.9% (0.02%) E200:
	TI	NS	NS

				PI	NO: ↓36.13% (0.01E) ↓43.1% (0.02%) WO: NS	NS		
**Polyphenols**	0.01+100 mg/kg of VitE (0.01E), 0.02,	E, 100 mg/kg (E100), 200 mg/kg (E200)			2 control, without (NG) or with grain (WG) contaminated		35	Mazur-Kusnirek et al. (2019c)
			Antioxidant status in BM	Vitamin C	NG: ↓12.07% (0.01E) ↓23.52% (0.02%)	E100: ↓12.97% (0.01E) E200: ↓18.37% (0.01E) ↓29.01% (0.02%)		
				Retinol	NG: NS WG: ↑32.98% (0.01E) ↑68.09% (0.02%)	NS		
				Total tocopherols	NG: ↑128.82% (0.01E) WG: ↑495.46% (0.01E)	E100: ↓60.47% (0.02%) E200: ↓30.32% (0.01E) ↓61.07% (0.02%)		

	Vitamin E equivalent	NG: ↑203.57% (0.01E) WG: ↑493.02% (0.01E)	E100: ↓37.96% (0.01E) ↓65.69% (0.02%) E200: ↓38.7% (0.01E) ↓66.11% (0.02%)
	TBARS	NG: NS WG: ↓55.26% (0.01E) ↓42.63% (0.02%)	NS
Carcass trait, structure of the gastrointestinal tract and digesta pH	Carcass dressing percentage	NG: ↓2.57% (0.01E) ↓3.84% (0.02%) WG: NS	NS
	Liver [%]	NG: ↑26.97% (0.02%)	NS
	BM, heart, Abdominal fat	NS	NS
	Crop, proventricolous, gizzard weight, digest pH	NS	NS
	Small intestine length	NG: ↑22.28% (0.01E) ↑24.17% (0.02%)	NS

Small intestine weight	NG: ↑20.65% (0.01E) ↑25.54% (0.02%)	E100: ↓9.76% (0.01E)
Small intestine digesta pH	NS	NS
Caeca length	WG: ↓13.25% (0.01E)	NS
Ceca weight, digesta pH	NS	NS

Abbreviations: AI, atherogenicity index, BM, breast muscle; CY, carcass yield; CW, carcass weight; DPPH, 1,1-diphenyl-2-picrylhydrazyl radical scavenging activity; DFAs, neutral and hypocholesterolemic Fatty Acids; ENB, Enterobacteriaceae; FAC, fatty acid profile; HT, heat treated tissue; IL, ileotibalis muscle; LAB, lactic acid bacteria; MDA, malondialdehyde; MUFA, monounsaturated fatty acids; NS, not significative; OFAs, hypercholesterolemic fatty acids; ORACFL, antioxidant capacity α-tocopherol content; PI, peroxidisability index; PM, pectoralis major (muscle); PSY, psychrophilic bacteria; PUFA, polyunsaturated fatty acids; RLE, relative length; RW, relative weight;; TBARs, thiobarbituric acid reactive substances; TI, thrombogenicity index, TM, thigh meat; TPC, total phenolic content; TVC, total viable counts (bacteria); UFA, unsaturated fatty acids; UP, unidentified peaks. (↑—increase; ↓—decrease).

Table S2. Effects of different plant feed additives on quantitative and qualitative traits of eggs production.

Plant extract	Dose extract	Vitamin evaluated	Type of parameter	Trait evaluated	Comparison to negative control: effect (plant product dose)	Comparison to positive control: effect (dose)	Period of study	Reference
**Rosemary extract**	0.05%, 0,1%	E, 200 mg/kg	Fatty acids composition of fresh eggs; Lipid oxidative stability of omega3-fatty acid (FA)-enriched eggs: Lipid hydropeorxides, TBARS	FAC fresh eggs	NS	NS	25 days	(Galobart et al 2001)
			Lipid oxidation markers lipid hydroperoxide, MDA; Fatty acid profile; α-tocoherol content, susceptibility to iron-induced lipid oxidation		NS	NS		
**Rosemary plant (RP), oregano (OP), saffron (SAF)**	Respectively 0.5%,0.5%, 2%	E, 200 mg/kg	Egg quality traits and production; Lipid oxidation (MDA)	Yolk colour	NS	↑7.84% (SAF)	56 d	(Botsoglou et al., 2005)

				Lipid oxidation (MDA)	↓ SAF>RP=OP ($P<0.05$)	↑ OP=RP=SAF ($P<0.05$)		
**Rosemary (RP)**	0.5% (0.5RP), 1% (1RP)	E, 200 mg/kg	Egg production, egg quality traits. Lipid oxidation.	Lipid oxidation of eggs (MDA)	↓ 0.5RP>1RP	↑ 0.5RP>1RP	60 d	(Florou-Paneri et al., 2006)
				Lipid oxidation of yolks	↓RP (0.5%)> RP(1%)	↑0.5RP>1RP		
**Olive leaves**	0.01	E, 200 mg/kg	Fatty acid composition, MDA in fresh and stored *n-3* enriched eggs (60 d)	Only in stored eggs			33 d	(Botsoglou et al., 2013)
				C18:1 oleic	↓3.87%	NS		
				C18:2 (n-6) linoleic	↑6.1%	NS		
				C22:6 (n-3) DHA	↑14.94%	NS		
				MUFA	↓4.1%	NS		
				PUFA	↑7.64%	NS		
				n-6 PUFA	↑5.77%	NS		
				n-3 PUFA	↑11.99%	NS		
				Ratio n-6/n-3	↓5.6%	NS		
				Lipid hydroperoxide levels	↓ ($P<0.05$)	NS		
				α-tocopherol in eggs stored (40 d)	↑57.4%	NS		

				α-tocopherol in eggs stored (60 d)	NS	↓73.2%		
**Olive leaves**	0.5% (0.5OL), 1% (1OL)	E, 200 mg/kg	Lipid oxidation: lipid hydroperoxide, MDA; Fatty acid profile; α-tocopherol content, susceptibility to iron-induced lipid oxidation	Cumen hydroperoxides	↓88.96% (0.1OL)	↑100.5 % (0.5OL)	33 d	(Botsoglou et al., 2013)
				MDA after iron induced oxidation	↓1OL	↑0.5OL>1OL		
**Green tea (powder, GTP; extract,GTE; marigold (powder,MP; extract, ME)**	0.5, 1.5	E, 200 mg/kg	Egg production, Egg quality, egg yolk cholesterol	Egg mass	NS	↓11.1% (MP)	12 weeks	(Ariana et al., 2011)
				Yolk weight	NS	↓4.61% (GTE, GTP)		
				CHOL/yolk	↓2.67% (MP) ↓7.47% (ME) ↓6.1% (GTP) ↓8% (GTE)	↑7.13% (MP) ↑1.85% (ME) ↑3.36% (GTP) ↑1.26% (GTE)		

				CHOL/egg	↓8.26% (ME) ↓12.1% (GTE)	NS		
				TG/yolk	↓4.19% (ME) ↓ 3.41%(GTP) ↓5.16% (GTE)	NS		
**Roselle Calyx**	Crude extracts (1,2%), Powder (2,4%)	E, 250 mg/kg	Egg production, Egg weight, Specific gravity, Haugh unit, ph of yolk and albumin; TBARs in yolk;TBARs in refrigerator and room temperature	None			8 weeks	(Sukkhavanit et al., 2011)
**Anise seed (quails)**	1% (1AS), 2% (2AS)	E, 600 mg/kg	Egg production, Egg quality parameters, yolk colour	Yolk colour			29 days	Christaki et al., 2011
				L*	NS	↑3.75% (1AS) ↑3.42% (2AS)		
				a*	NS	↓50.3% (1AS) ↓36.3 %(2AS)		
				b*	NS	NS		

**Sage extract (SE)**	2.5	E, 30 mg/kg	Egg production	Number of eggs produced/hen, g/egg, g/egg per hen per d, g egg/g feed intake2	NS	NS	28	Loetscher et al. (2014)

Abbreviations: CHOL, cholesterol; FAC, fatty acid composition; MDA, malondialdehyde; NS, not significative; PFA, plant feed additive; TBARs, thiobarbituric acid reactive substances. (↑—increase; ↓—decrease).
